# Ice-Templated and Cross-Linked Xanthan-Based Hydrogels: Towards Tailor-Made Properties

**DOI:** 10.3390/gels9070528

**Published:** 2023-06-29

**Authors:** Irina Elena Raschip, Nicusor Fifere, Maria Marinela Lazar, Gabriela-Elena Hitruc, Maria Valentina Dinu

**Affiliations:** “Petru Poni” Institute of Macromolecular Chemistry, Grigore Ghica Voda Alley 41A, 700487 Iasi, Romania; fifere.nicusor@icmpp.ro (N.F.); maria.lazar@icmpp.ro (M.M.L.); gabihit@icmpp.ro (G.-E.H.); vdinu@icmpp.ro (M.V.D.)

**Keywords:** xanthan, cross-linking, ice templating, stabilization, controlled swelling, porosity

## Abstract

The use of polysaccharides with good film-forming properties in food packaging systems is a promising area of research. Xanthan gum (XG), an extracellular polysaccharide, has many industrial uses, including as a common food additive (E415). It is an effective thickening agent, emulsifier, and stabilizer that prevents ingredients from separating. Nevertheless, XG-based polymer films have some disadvantages, such as poor mechanical properties and high hydrophilic features, which reduce their stability when exposed to moisture and create difficulties in processing and handling. Thus, the objective of this work was to stabilize a XG matrix by cross-linking it with glycerol diglycidyl ether, 1,4-butanediol diglycidyl ether, or epichlorohydrin below the freezing point of the reaction mixture. Cryogelation is an ecological, friendly, and versatile method of preparing biomaterials with improved physicochemical properties. Using this technique, XG-based cryogels were successfully prepared in the form of microspheres, monoliths, and films. The XG-based cryogels were characterized by FTIR, SEM, AFM, swelling kinetics, and compressive tests. A heterogeneous morphology with interconnected pores, with an average pore size depending on both the nature of the cross-linker and the cross-linking ratio, was found. The use of a larger amount of cross-linker led to both a much more compact structure of the pore walls and to a significant decrease in the average pore size. The uniaxial compression tests indicated that the XG-based cryogels cross-linked with 1,4-butanediol diglycidyl ether exhibited the best elasticity, sustaining maximum deformations of 97.67%, 90.10%, and 81.80%, respectively.

## 1. Introduction

Polysaccharides are attractive and promising natural polymers for the development of sustainable biomaterials with a wide range of applications. Their origins are plant/seed (cellulose, starch, and pectin), bacterial (dextran, xanthan gum, gellan gum, and salecan), seaweeds (alginate, ulvan, and carrageenan), or marine and terrestrial animal by-products (chitin, hyaluronic acid, and chondroitin sulfate) [1,2,3,4,5,6,7,8,9,10]. Polysaccharides possess unique properties including biocompatibility, biodegradability, and lack of toxicity that make them suitable for multiple applications [1,2,3,4,5,6,7,8]. For instance, some polysaccharides with hydrophilic groups (-OH or -COOH), such as dextran, xanthan gum, or alginate, have good water solubility and can form hydrogen bonds with water molecules, which support their suitability as thickening agents to increase the viscosity of the formulation and improve its uniformity [1,2,3,6]. On the other hand, polysaccharides modified with hydrophobic groups, such as fatty acid esters, can be used as emulsifying agents, stabilizing emulsions or suspensions [1,2,3,6]. Similarly, some polysaccharides could be utilized as binding agents to the uniformity of the formulations, while others could be applied as coating agents protecting the bioactive compound from degradation or controlling its release over time [1,2,3,5]. Currently, researchers are exploring different strategies for modifying and functionalizing polysaccharides to further tailor their properties for specific applications, such as drug delivery systems [1,2,3], wound dressings [3,4], tissue engineering [3,4,5], food packaging [6], and environmental protection [2,7,8].

Xanthan gum (XG) is a hydrophilic, non-toxic, biodegradable, and biocompatible anionic polysaccharide produced during the fermentation process of a Gram-negative bacterium, namely *Xanthomonas campestris* [11,12]. XG consists of (1,4)-β-D-glucose backbone with side chains of (1,3)-α-linked D-mannose-(1,2)-β-D-glucuronic acid-(1,4)-β-D-mannose on alternating residues [11,12,13]. XG also contains approximately 4.7% of acetyl groups and 3.5% pyruvic acid and forms high-viscosity and stable pseudoplastic dispersions over a wide variety of pH values as well as in the presence of various salts [14]. These properties make XG an important additive in a wide range of industries, including food, pharmaceuticals, and personal care products [6,11,12,13]. Moreover, a broad variety of biomaterials based on XG in the form of coatings, films, nanoparticles, or hydrogels have been prepared for multiple applications including food packaging, environmental protection, and drug delivery [11,13]. However, the uncontrolled swelling and rapid degradation upon storage are two major drawbacks of XG. To overcome these disadvantages and enhance the physicochemical properties and functional features of XG, hydrogels consisting of XG and various natural (chitosan [15,16], N-trimethyl chitosan [17], carboxymethyl chitosan [15], carboxymethyl cellulose [18], alginate [19], starch [20], lignin [21,22]) or synthetic polymers (polyvinylalcohol (PVA) [19,23,24,25], polyvinylpyrrolidone (PVP) [26], polyethyleneglycol (PEG) [27], poly(acrylic acid) (PAA) [20,26,27]) have been developed.

XG-based networks with improved mechanical properties have been also obtained when solutions of XG were subject to successive cycles of freezing and thawing [28]. The manufacturing process known as cryogelation or cryostructuring allows biopolymers to change their features as a result of controlled and repeated freeze–thawing cycles [28,29]. This means that the modification occurring in the polymeric materials are mainly driven by the cryogenic treatment (rate of freezing, gel preparation temperature, duration of freezing, the number of freeze–thawing cycles, rate of thawing, etc.) [2,23,28,29,30,31,32,33,34]. The materials obtained through this ice-templating process are called cryogels, and they possess interconnected macropores with shapes and sizes tailored by the nature and concentration of the polymer and the freeze–thawing conditions [2,23,32,33,34]. Furthermore, cryogels exhibit enhanced mechanical properties compared to conventional hydrogels due to the peculiarity of the cryogenic process, in which the network walls are formed by the cryo-concentrated polymer [2,31,32,33,34]. The unique structural features of the cryogel-type materials that prevent clogging support their application for the separation of proteins, plasmids cells (mammalian, bacterial, and yeast), and viruses [35,36,37,38,39,40]. Currently, various polysaccharide-based cryogels have gained significant attention within academia and industry due to their environment friendliness, low cost, and multiple functionalities [2,41,42,43,44,45]. Thus, polysaccharide-based cryogels have found applications in a variety of fields, including drug delivery, tissue engineering, the food and beverage industry, and wastewater treatment [2,41,42,43,44,45].

However, during the subsequent thawing and removal of the ice crystals, the biopolymer structure may be weakened, leading to structural collapse or deformation. To prevent this, chemical cross-linking is often used to stabilize the biopolymer structure and maintain its integrity during the freeze-thawing process [36,39,45]. The cross-linking rate is an important parameter to consider when designing cryogels, as it can affect the mechanical properties, porosity, and stability of the resulting material. Therefore, optimizing the cross-linking rate is crucial to achieving robust cryogels. If the cross-linking rate is too fast, the polysaccharides may cross-link and aggregate before the ice crystal formation occurs, resulting in the formation of conventional hydrogels or particles rather than cryogel-type materials. This can lead also to the formation of a dense and compact structure, which does not have the desired properties. On the other hand, if the cross-linking rate is too slow or insufficient, the cryogels may exhibit poor mechanical performance, as the biopolymer structure was not adequately stabilized. This can result in cryogel-type materials that are weak, fragile, and prone to deformation or collapse. Therefore, it is important to optimize the cross-linking rate to achieve a balance between structural stability and appropriate properties. The optimization of the cross-linking rate depends on several factors, such as the type and concentration of cross-linking agent, the pH and temperature of the reaction, as well as on the duration of the reaction [46]. Different cross-linking agents may have different reaction rates and mechanisms, which can affect the characteristics of the resulting cryogel. By carefully controlling the cross-linking rate during cryogel fabrication, the overall performance and functionality of cryogels can be improved for a wide range of applications, including drug delivery, tissue engineering, biocatalysis, food packaging, and environmental protection. In this regard, the aim of this study was to investigate the chemical cross-linking of XG with glycerol diglycidyl ether, 1,4-butanediol diglycidyl ether, or epichlorohydrin under cryogelation conditions. The objectives followed in this work were: (i) to design cross-linked xanthan gum-based materials with improved physicochemical features below the freezing point of the reaction mixture; (ii) to obtain the optimum reaction conditions for preparation of XG-based cryogels by changing the type and amount of cross-linker (glycerol diglycidyl ether (R1), 1,4-butanediol diglycidyl ether (R2), or epichlorohydrin (R3)), XG concentration, and gel preparation temperature; and (iii) to characterize the cross-linked XG-based cryogels using FTIR, SEM, AFM, swelling kinetics, and compressive tests.

## 2. Results and Discussion

### 2.1. Preparation of XG-Based Cryogels as Microspheres, Monoliths, and Films

**Figure 1:**
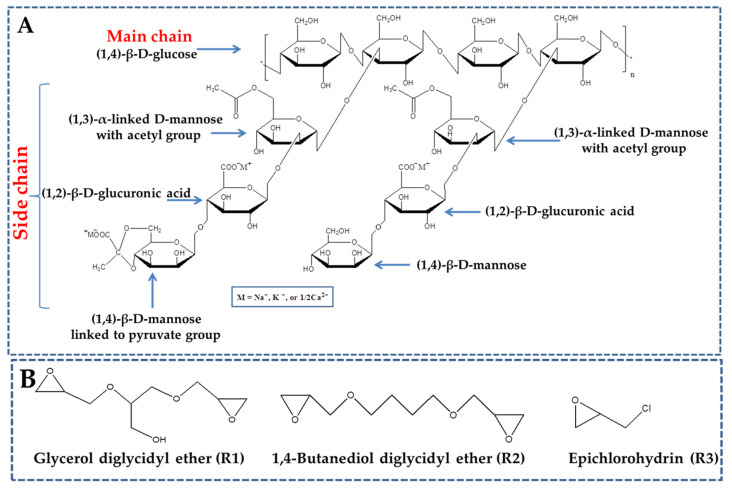
(**A**) Schematic representation of XG chemical structure; (**B**) chemical structures of the cross-linkers used to prepare the cryogel biomaterials.

**Figure 2:**
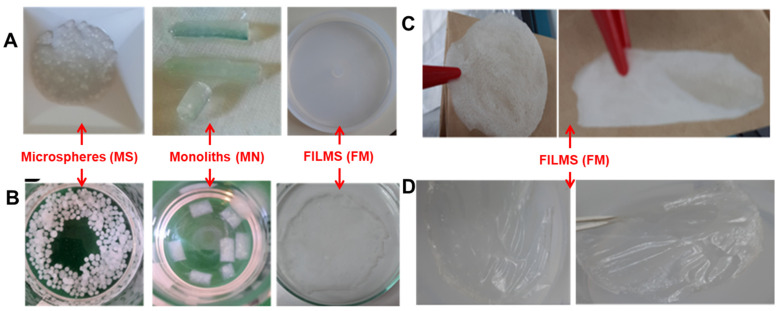
Optical microscopy images showing the appearance of XG-based cryogels in the form of MS, MN, and FM at different stages of the preparation (**A**), purification (**B**), and drying process either by lyophilization (**C**) or in an oven at 40 °C (**D**).

In the current research, we prepared new cryogel-type biomaterials based on XG using glycerol diglycidyl ether (R1), 1,4-butanediol diglycidyl ether (R2), and epichlorohydrin (R3) as cross-linkers through cryogelation (Figure 1). The use of different cross-linkers and concentrations allowed us to optimize the degree of cross-linking and tailor the properties of the resulting cryogels. In addition, the preparation of XG-based cryogels in form of microspheres (MS), monoliths (MN), and films (FM) (Figure 2) enabled the evaluation of the effect of cryogelation on different morphologies of the biomaterials.

In principle, the synthesis of XG matrices followed the same basic steps, with slight modifications depending on the presentation form of the sample (MS, MN, or FM). The cross-linking reactions took place in an alkaline environment using a 2M solution of sodium hydroxide (NaOH). The first step involved preparing a 1% solution of XG in deionized water. Then, various ratios of cross-linkers (glycerol diglycidyl ether, 1,4-butanediol diglycidyl ether, or epichlorohydrin) were added to the XG solution to obtain polymer matrices with different degrees of cross-linking. All stages of the synthesis were carried out in an ice bath to reduce the rate of the cross-linking reaction and maintain the fluidity of the mixture for subsequent processing. The cross-linker ratios used in the study were expressed in volumetric amounts corresponding to 100 mL of XG and NaOH solution mixture (Table 1). The reaction time in the ice bath is relatively short, typically on the order of minutes, after which the sub-cooled mixture was processed according to the desired form of presentation of the biomaterial, as follows:

- To obtain the XG cryogel microspheres, the polymer/cross-linker mixture was introduced into 5 mL syringes equipped with a needle and then dropped into liquid nitrogen (LN, −196 °C). The rapid cooling process in LN causes the droplets to solidify into spherical particles, whose size depends on the diameter of the syringe needle. After removing the microspheres from the LN, they were transferred to a freezer at a temperature of −20 °C for 24 h to complete the cross-linking process (Figure 2).
- To obtain XG cryogel monoliths, the polymer/cross-linker mixture was introduced into syringes with a diameter of 4 mm, and then the syringes were transferred to a freezer at a temperature of −20 °C for 24 h. During this time, the cross-linking reaction is completed and XG cryogels of a tubular shape are obtained (Figure 2).
- To obtain XG cryogel films, the sub-cooled reaction mixture was poured into Teflon Petri dishes and then transferred to a freezer at a temperature of −20 °C for 24 h (Figure 2).

After 24 h, all samples were removed from the freezer and transferred in frozen state to lyophilizer for drying. After drying, all samples were purified via extensive washing with distilled water (Figure 2). Finally, the samples were dried again either by lyophilization or in an oven at 40 °C. Sample code, composition, gel fraction yield (GFY, %), and the type form of XG-based hydrogels are shown in Table 1.

As shown in Table 1, an increase in cross-linker concentration led to an increase in GFY values, as expected. Furthermore, the GFY values depended on the type of cross-linker and the form of the XG-based cryogels, i.e., microspheres, monoliths, or films. For XG-based cryogels in microsphere form, glycerol diglycidyl ether was the most effective cross-linking agent at a concentration of 7 *v/v*%, resulting in a GFY value of 87.87%. In the case of XG-based cryogels as monoliths or films, 1,4-butanediol diglycidyl ether was the most efficient cross-linker, conducting to GFY values of 83.4% and 98.8%, respectively. However, the highest GFY values were obtained when XG-based cryogels were prepared as films irrespective of cross-linker type.

### 2.2. FTIR Analysis

**Figure 3:**
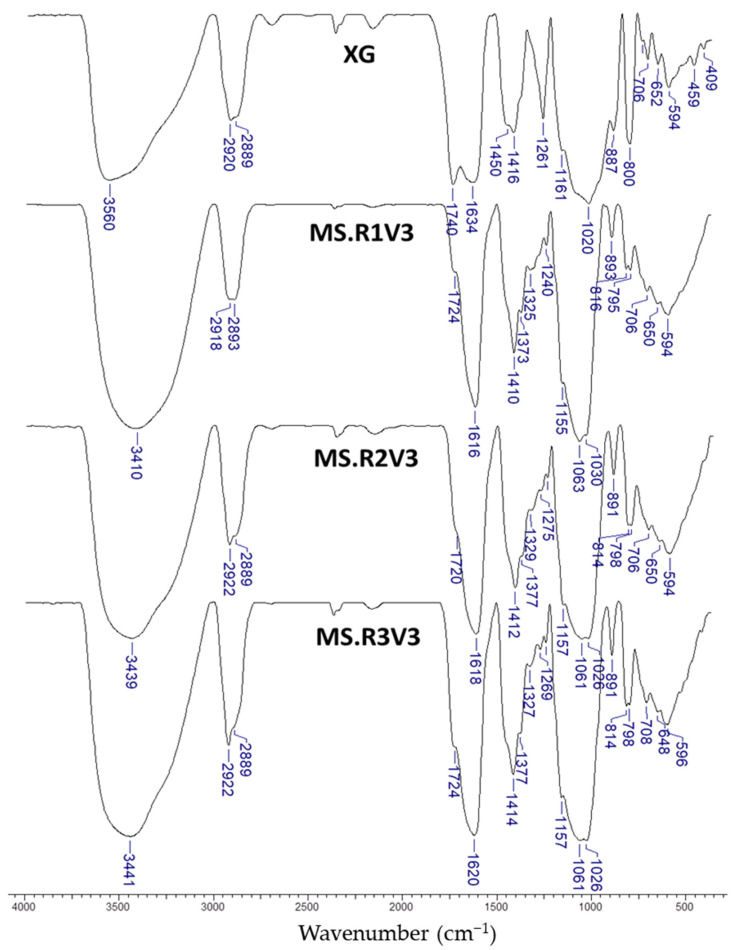
FTIR spectra corresponding to XG and networks based on XG obtained via cross-linking with R1, R2, or R3.

To identify the chemical modifications achieved via XG cross-linking, FTIR spectroscopy was used first as a fast characterization tool. The FTIR spectrum of XG powder was compared with the FTIR spectra of XG networks obtained by cross-linking with R1, R2, or R3 (samples MS.R1V3, MS.R2V3, and MS.R3V3, respectively, Figure 3).

In the FTIR spectrum of XG the following absorption peaks are visible: a broad absorption peak at 3560 cm^−1^ corresponding to the stretching vibration of -OH groups; two peaks at 2920 and 2889 cm^−1^ assigned to the asymmetric and symmetric vibration of -CH_2_ groups; two characteristic peaks at 1740 cm^−1^ and 1634 cm^−1^ attributed to the stretching of carbonyl (C=O) of the acetyl groups and asymmetrical stretching of C=O group of pyruvate groups, respectively [47,48,49,50]; an absorption peak at 1416 cm^−1^ corresponding to the symmetric stretching vibration of carboxylate (-COO−) groups [47,51]; the shoulder at 1161 cm^−1^ and the broad peak at 1020 cm^−1^ referred to the C-O stretching vibration in secondary alcohol and to the stretching vibration of the C-O-C bridge in anhydroglucose (AGU) units, respectively [23,48,49,50]. All these peaks were also identified in the FTIR spectra of XG networks obtained via cross-linking (samples MS.R1V3, MS.R2V3, and MS.R3V3, Figure 3) with some blue- or red-shifts, as follows: the peak at 3560 cm^−1^ in the FTIR spectrum of XG powder corresponding to the -OH stretching vibration were blue-shifted to 3410, 3439, and 3441 cm^−1^ in the FTIR spectra of MS.R1V3, MS.R2V3, and MS.R3V3 networks, respectively. The intensity of the band from 1740 cm^−1^, characteristic to the stretching of carbonyl (C=O) of the acetyl groups, decreased and experienced a hypsochromic shift to 1724 cm^−1^ and 1720 cm^−1^ in the XG cross-linked matrices due to the hydrolysis reaction, which easily occurs in alkaline conditions [52]. The band at 1634 cm^−1^ corresponding to asymmetrical stretching of C=O group of pyruvate groups was blue-shifted at 1616, 1618, and 1620 cm^−1^ in the FTIR spectra of MS.R1V3, MS.R2V3, and MS.R3V3 networks, respectively. The characteristic absorption peak at 1020 cm^−1^ in the FTIR spectrum of XG powder corresponding to the stretching vibration of the C-O-C bridge in AGU units was red-shifted to 1030 cm^−1^ and 1026 cm^−1^ in the FTIR spectra of MS.R1V3, MS.R2V3, and MS.R3V3 networks, respectively. In addition, the presence of clear peaks at 1063 and 1061 cm^−1^ in the FTIR spectra of the XG cross-linked matrices indicates the appearance of supplementary ether (–C–O–C–) and hydroxyl (-OH) groups [23,48,49,50,51,53]. All these changes support the efficient occurrence of the cross-linking reaction performed with all cross-linkers.

### 2.3. Internal Morphology

**Figure 4:**
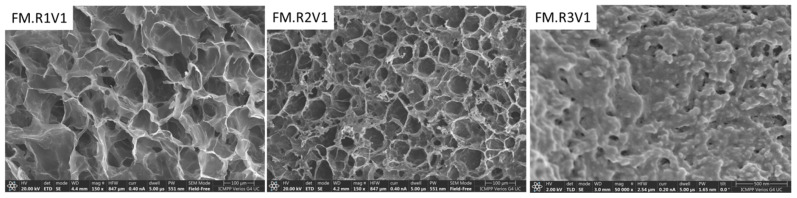
SEM micrographs of cross-sections through the XG-based cryogels prepared as FM using R1, R2, or R3 at a 0.7 *v/v*% ratio. The film’s thickness was about 0.5 mm.

**Figure 5:**
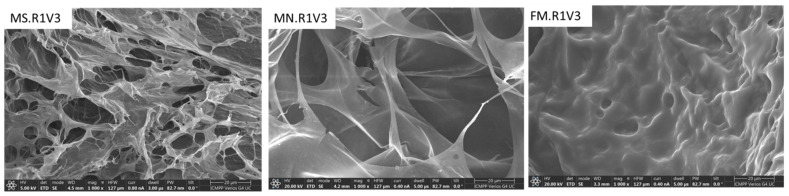
SEM micrographs of cross-sections corresponding to XG-based cryogels prepared in the form of MS, MN, and FM using cross-linker R1 in a 7 *v/v*% ratio.

**Figure 6:**
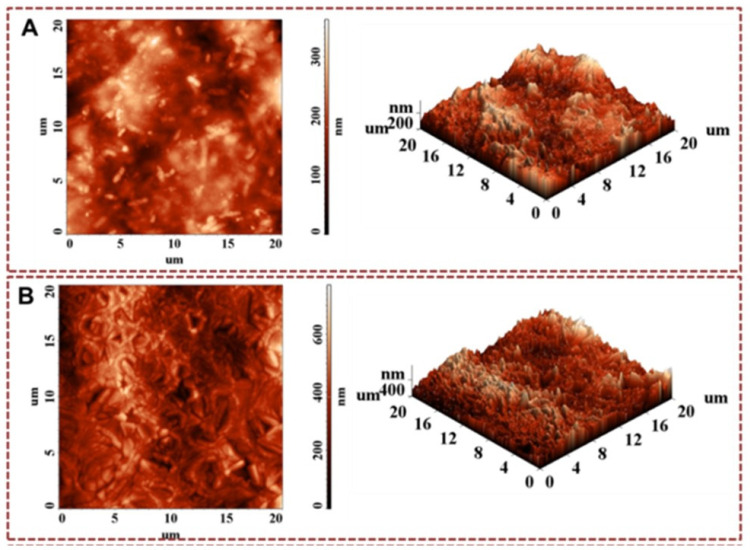
AFM images of XG-based networks in film form: (**A**) FM.R3V1 and (**B**) FM.R3V3.

The morphology studies of the obtained XG-based networks were carried out by evaluating the porosity, texture, and roughness of the surfaces through SEM (Figure 4 and Figure 5) and AFM (Figure 6) analysis.

Figure 4 shows a heterogeneous morphology with interconnected pore characteristic of gels obtained via cryogelation. The average pore size depends on both the nature of the cross-linker and the cross-linking ratio. The use of a larger amount of cross-linker leads both to a much more compact structure of the pore walls and to a significant decrease in the average pore size (Figure 4 and Figure 5 make a comparison between samples FM.R1V1 and FM.R1V3, respectively). The morphology of XG-based networks can also be controlled by the form of presentation of the material—MS, MN, or FM (Figure 5). Thus, if a porous structure is desired, the samples can be synthesized in the form of MS (Figure 5, sample MS.R1V3), and if a compact structure is desired, XG-based networks can be prepared in the form of films (Figure 5, sample FM.R1V3). The FM.R1V3 cryogel lacks pores, suggesting that the inherent nature of the cryogel itself is responsible for the absence of pore formation.

The morphology of the XG-based networks obtained in the form of films was also examined by AFM analysis (Figure 6). AFM images were obtained using an SPM Solver PRO-M-type apparatus (NT-MDT Co. Zelenograd, Moscow, Russia) that uses a high-resolution cantilever of the “Golden” type (NSG10/Au/50 silicon coated with a conductive layer of Au). The topographic images (Figure 6) were obtained in the intermittent scanning regime and were repeated on several areas of the same sample. By analyzing the roughness values in Table 2 as well as the AFM images (Figure 6A,B), it is observed that the chemically cross-linked films in cryogenic conditions present a structured morphology.

The existence of structuring indicates that the orientation of macromolecules in the freezing phase is restricted by the centers of chemical cross-linking due to the decrease of the average free volume of the chain segments. The structured phase is stabilized upon thawing by the existence of the strengthening of physical bonds, so the morphology obtained by the orientation of the macromolecules in the freezing process is partially preserved at room temperature.

The comparative analysis of two films with different degrees of cross-linking (FM.R3V1 and FM.R3V3) shows that in the sample with a lower degree of cross-linking (sample FM.R3V1, cross-linker content of 0.7 *v/v*%), reduced structuring with a morphology in the form of rods with random distribution appears. In the sample FM.R3V3, in which the degree of cross-linking is much higher (7 *v/v*%), an advanced structuring appears with a floral appearance, and the average surface roughness is almost double that of the sample FM.R3V1 (Figure 6). The increase in the degree of organizational structuring in the sample with higher cross-linking density shows the cross-linking effect on the polymer by decreasing the molecular mass of the polymer chains between two neighboring cross-linking centers. The decrease in the mobility of the macromolecular chains by increasing the cross-linking density in the FM.R3V3 sample led to a much more complex supramolecular morphology. This effect was determined by the process of orientation and stabilization by physical bonds of the macromolecular chains of XG in freezing conditions with the increase in the density of physical interactions as an effect of the proximity of the chemically cross-linked macromolecular chains.

### 2.4. Mechanical Properties

**Figure 7:**
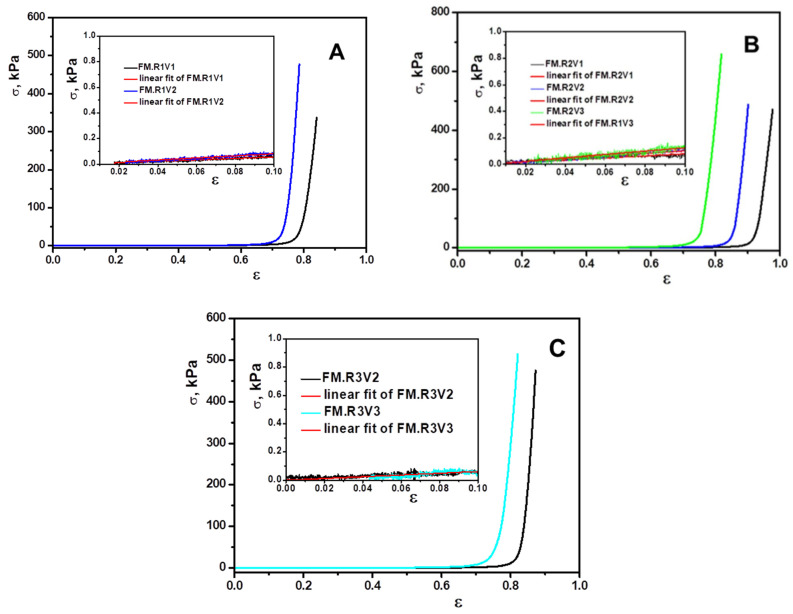
Stress–strain curves recorded for XG-based cryogels obtained by cross-linking with glycerol diglycidyl ether (**A**), 1,4-butanediol diglycidyl ether (**B**), and epichlorohydrin (**C**).

The strength and uniaxial compression tests on the cryogel samples were performed using Shimadzu mechanical testing equipment (EZ-LX/EZ-SX Series) using the parallel plate measuring system. Wet samples of approximately 5–8 mm length, 6–8 mm width, and 4–5 mm height were placed between the plates, and to ensure complete contact between the sample and the plate surface, an initial force of 0.02 N was applied. The imposed strain rate was 0.2 mm/min, and the applied force was fixed at 100 N. The uniaxial compressive stress (σ, N/m^2^) and strain (ε) were determined according to a previously presented method [21]. The mechanical properties of XG-based cryogels were evaluated, following the influence of the degree of cross-linking and the type of cross-linker. The stress-strain profiles registered for these biomaterials are shown in Figure 7.

As can be seen from the stress–strain curves registered for the XG-based cryogels, the addition of a higher content of cross-linker induces a stiffening of the matrix due to the creation of more cross-linking points. XG-based cryogels cross-linked with 1,4-butanediol diglycidyl ether (R2) showed the best elasticity, sustaining a maximum deformation of 97.67%, 90.10%, and 81.80%. The cryogels obtained with R2 showed the highest compressive strength with values between 470.58 kPa and 658.84 kPa (Figure 7, Table 3). The modulus of elasticity was calculated from the slope of the linear dependence of the stress–strain curves at a strain between 0 and 10%, and the results obtained are included in Table 3. The values of the modulus of elasticity increased with the increase of the cross-linker content in the samples.

### 2.5. Swelling Properties

**Figure 8:**
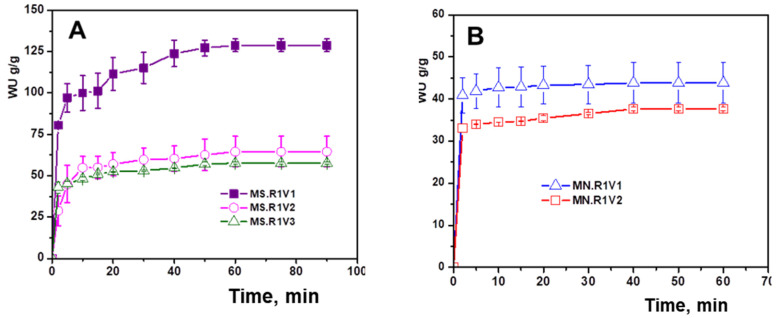

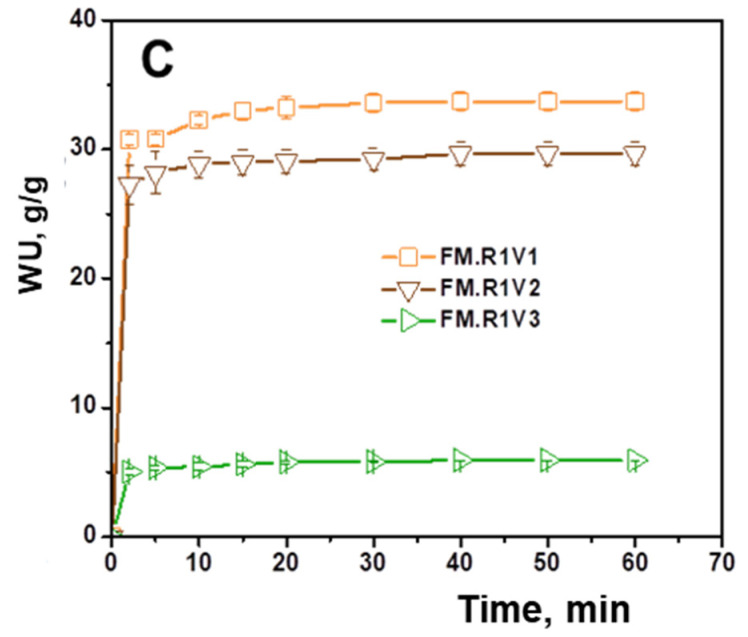
Kinetics of swelling in water of polymeric networks based on XG cross-linked with glycerol diglycidyl ether (R1) synthesized in the form of MS (**A**), MN (**B**), and FM (**C**).

The capacity of XG-based polymer networks to swell in aqueous medium was determined by the gravimetric method (Figure 8). The swelling behavior of the cryogels was evaluated by immersing the dry samples (approx. 0.01 g) in distilled water and weighing them at well-established time intervals.

The swelling kinetics of polymer networks based on XG are influenced by a number of factors, such as chemical composition and types of components; their sensitivity to changes in the analysis environment; the presence of pores, their distribution, and diameter; and the specific contact area with the environment. From the swelling profiles shown in Figure 8, it can be clearly observed that the cross-linking ratio and the presentation form of the XG matrix has a major influence on the water retention capacity. Thus, it was highlighted that with the increase in the amount of cross-linking agent, the polymer matrix absorbs less water as a result of the increase in the cross-linking density. In addition, the glycerol diglycidyl ether cross-linked XG-based polymer networks synthesized as films (Figure 8C) retained the smallest amount of water due to the much more compact pore wall structure as observed via SEM microscopy analysis (Section 2.3, Figure 5).

## 3. Conclusions

Ice-templated hydrogels based on XG were successfully prepared regardless of the cross-linking agent used.

The XG-based materials exhibited a heterogeneous morphology with interconnected pores characteristic of gels obtained by cryogelation; the average pore size depends on both the nature of the cross-linker and the cross-linking ratio.

The influence of both the type and amount of cross-linker and the form of presentation of the matrix (MS, MN, or FM) on the degree and kinetics of swelling was observed; the glycerol diglycidyl ether cross-linked XG-based polymer networks synthesized as films retained the lowest amount of water due to the much more compact pore wall structure.

The addition of a higher content of cross-linker induces a stiffening of the matrix, due to the generation of more cross-linking points. In addition, 1,4-butanediol-diglycidyl-ether-cross-linked XG-based polymer networks exhibited the highest elasticity.

Our results highlight that XG-based polymer networks with tailored properties in terms of mechanical strength, porosity, and swelling capacity could be properly developed by conducting the chemical cross-linking by cryogelation approach. This process allows for the preservation of the porous structure, which is advantageous for applications requiring high porosity and interconnected pore networks. The porosity of the XG-based cryogels was adjusted by modifying the nature of the cross-linker and the cross-linking ratio, providing control over the diffusion of substances through the material. This property is particularly relevant for applications like controlled drug delivery systems, where precise regulation of the release kinetics is required. The swelling capacity of the XG-based cryogels was controlled by the degree of cross-linking. This feature is crucial in tissue-engineering applications, as it allows for the absorption of water and nutrients, mimicking the natural environment required for cell growth and tissue regeneration. These materials offer versatility and can be tailored to meet the specific requirements of various applications, ranging from controlled drug delivery and tissue engineering to food packaging and separation processes. Further research and development in this area can lead to advancements in these fields and contribute to the design of novel functional materials.

## 4. Materials and Methods

### 4.1. Materials

XG with an average molecular weight of 1.98 × 10^6^ g/mol was purchased from Sigma-Aldrich (St. Louis, MO, USA) and used as received. Glycerol diglycidyl ether (R1), 1,4-butanediol diglycidyl ether (R2), and epichlorohydrin (R3) were also purchased from Sigma-Aldrich and used as cross-linkers.

### 4.2. Methods

#### 4.2.1. Synthesis of XG-Based Hydrogels

XG-based hydrogels were prepared as microspheres, monoliths, and films by cross-linking XG with various cross-linkers below the freezing point of the reaction solution at −20 °C. The parameters varied in the preparation of XG cryogels were the type of cross-linker, the concentration of cross-linker, and the shape of the final material. Glycerol diglycidyl ether, 1,4-butanediol diglycidyl ether, and epichlorohydrin were used as cross-linkers in a concentration ranging from 0.7 *v/v*% to 7 *v/v*% (Table 1). An XG solution with a concentration of 1 wt% was first prepared. After that, each 5 g of XG solution (1%) was mixed with 1 mL of NaOH solution (2M) and the corresponding amount of cross-linker. The mixed solutions were cooled to 0 °C in an ice bath and kept under rigorous stirring about 10 min. Then, the mixtures were processed according to the desired form of presentation of the biomaterial. Thus, to prepare microspheres, the polymer/cross-linker mixture was introduced into 5 mL syringes equipped with a needle and then dropped into liquid nitrogen (LN, −196 °C). To obtain XG cryogel as monoliths, the polymer/cross-linker mixture was introduced into syringes with a diameter of 4 mm, while to synthesize XG cryogel as films, the sub-cooled reaction mixture was poured into Teflon Petri dishes. All samples were transferred to a freezer at a temperature of −20 °C for 24 h to complete the cross-linking irrespective of the shape of the final material. Thereafter, the samples were removed from the freezer and transferred in frozen state to lyophilizer for drying. After drying, all samples were purified by extensive washing with distilled water. Finally, the samples were dried again either via lyophilization or in an oven at 40 °C.

#### 4.2.2. Gel Fraction Yield (GFY, %)

GFY was calculated as ratio between the weight of dried XG hydrogels (*W_d_*) and the weight of all reactants involved in the cross-linking reaction (*W_m_*):

(1) $$GFY=\frac{W_{d}}{W_{m}}\times 100$$

#### 4.2.3. FTIR

The FTIR spectra of XG-based hydrogels were recorded with a Bruker Vertex 70 FTIR spectrophotometer (Bruker, Ettlingen, Germany) in the range 4000–400 cm^−1^, at a resolution of 2 cm^−1^, using the KBr pellet technique.

#### 4.2.4. Scanning Electron Microscopy (SEM)

The cross-sectional microstructure of the XG-based hydrogels was analyzed with a Quanta 200-FEI-type environmental scanning electron microscope (ESEM) (FEI Company, Hillsboro, OR, USA), at 20 kV in low vacuum mode. To reveal their internal morphology, a sharp blade was used to obtain cross-sections through the monoliths, microspheres, and films. The thickness of XG-based hydrogels as films was evaluated using a Vorel15240 stainless hardened digital caliper [54]. The results were reported as the mean value of five readings from different regions of each film.

#### 4.2.5. Atomic Force Microscopy (AFM)

The 2D and 3D surface images for the XG-based hydrogels were collected using a NTEGRA scanning probe microscope (NT-MDT Spectrum Instruments, Moscow, Russia) in atomic force microscopy (AFM) configuration. Rectangular silicon cantilevers NSG 03 (NT-MDT, Russia) with tips of height aspect ratio were used to scan the film surfaces. The AFM images and the surface parameters were processed and calculated using Nova v.19891 for Solver Software [55].

#### 4.2.6. Mechanical Tests

The uniaxial compression tests were performed on equilibrium-swollen hydrogels. A Shimadzu Testing Machine (EZ-LX/EZ-SX Series, Kyoto, Japan) was used to uniaxially compress the samples cut in form of the plates with a length of 5–8 mm, width of 6–8 mm, and height of 4–5 mm. A force of 100 N and a crosshead speed of 0.2 mm/min were used to analyze all samples. The strain (ε), the elastic modulus (G, kPa), and the compressive nominal stress (σ, kPa) were determined according to previously reported procedures [29,45].

#### 4.2.7. Water Uptake

The swelling kinetics of XG-based hydrogels was gravimetrically studied by soaking a defined amount of dried sample (0.01 g) in 10 mL aqueous solutions. The water uptake (*WU*, g/g) was calculated as:

(2) $$WU=\frac{W_{t}-W_{d}}{W_{d}},$$

where *W_t_* and *W_d_* are the weights of swollen hydrogels at time *t* and when dried, respectively.

## Figures and Tables

**Table 1:** Sample code, composition, GFY, and type form for XG-based cryogels.

Sample Code ^1^	Composition			^2^ GFY, %	Type Form
	XG, wt.%	Cross-Linker	C_crosslinker_, *v/v*%		
MS.R1V1	1	R1	0.7	78.80 ± 0.70	microspheres
MS.R1V2	1	R1	1.4	79.84 ± 0.77	microspheres
MS.R1V3	1	R1	7	87.87 ± 1.62	microspheres
MS.R2V1	1	R2	0.7	70.65 ± 0.26	microspheres
MS.R2V2	1	R2	1.4	74.72 ± 0.79	microspheres
MS.R2V3	1	R2	7	79.44 ± 1.09	microspheres
MS.R3V1	1	R3	0.7	80.10 ± 0.72	microspheres
MS.R3V2	1	R3	1.4	80.87 ± 0.66	microspheres
MS.R3V3	1	R3	7	80.91 ± 0.98	microspheres
MN.R1V1	1	R1	0.7	74.63 ± 0.76	monoliths
MN.R1V2	1	R1	1.4	77.38 ± 0.83	monoliths
MN.R1V3	1	R1	7	81.4 ± 1.87	monoliths
MN.R2V1	1	R2	0.7	77.29 ± 1.13	monoliths
MN.R2V2	1	R2	1.4	77.90 ± 1.06	monoliths
MN.R2V3	1	R2	7	83.4 ± 0.41	monoliths
MN.R3V1	1	R3	0.7	80.6 ± 0.43	monoliths
MN.R3V2	1	R3	1.4	81.2 ± 0.98	monoliths
MN.R3V3	1	R3	7	81.47 ± 1.45	monoliths
FM.R1V1	1	R1	0.7	93.28 ± 1.39	films
FM.R1V2	1	R1	1.4	93.80 ± 0.58	films
FM.R1V3	1	R1	7	94.88 ± 0.87	films
FM.R2V1	1	R2	0.7	89.37 ± 0.98	films
FM.R2V2	1	R2	1.4	90.43 ± 1.17	films
FM.R2V3	1	R2	7	98.8 ± 0.13	films
FM.R3V1	1	R3	0.7	90.12 ± 0.27	films
FM.R3V2	1	R3	1.4	90.64 ± 0.75	films
FM.R3V3	1	R3	7	96.79 ± 0.36	films

^1^ The sample code includes: MS, MN, or FM from the form of presentation of the biomaterial—namely MS; MN; or FM, R1, R2, or R3—indicates the cross-linking agent; R1: glycerol diglycidyl ether; R2: 1,4-butanediol diglycidyl ether; R3: epichlorohydrin; V1, V2, V3—represent the cross-linker content in *v/v*% in the reaction mixture; ^2^ gel fraction yield (GFY, %) was determined using the relationship in Equation (1).

**Table 2:** Root mean square.

Scanning Area	10 × 10 μm^2^	20 × 20 μm^2^	30 × 30 μm^2^	40 × 40 μm^2^
FM.R3V1	30.48	49.87	96.63	128.89
FM.R3V3	78.86	101.35	129.59	240.18

**Table 3:** Experimental data obtained for XG-based cryogels from strength and uniaxial compression tests.

Sample Code	Cross-Linker Type	C_cross-linker_, *v/v*%	Compressive Strength, kPa	Maximum Deformation, %	Elastic Modulus, kPa	R^2^
FM.R1V1	R1	0.7	336.91	84.11	0.562	0.985
FM.R1V2	R1	1.4	477.63	78.61	0.812	0.988
FM.R2V1	R2	0.7	470.58	97.67	0.798	0.958
FM.R2V2	R2	1.4	487.42	90.10	1.028	0.989
FM.R2V3	R2	7	658.84	81.80	1.248	0.969
FM.R3V2	R3	1.4	474.60	87.21	0.609	0.918
FM.R3V3	R3	7	514.37	82.03	1.262	0.990

## References

[1] Tudu M., Samanta A. (2023). Natural polysaccharides: Chemical properties and application in pharmaceutical formulations. Eur. Polym. J..

[2] Dragan E.S., Dinu M.V. (2020). Advances in porous chitosan-based composite hydrogels: Synthesis and applications. React. Funct. Polym..

[3] Tang W., Wang J., Hou H., Li Y., Wang J., Fu J., Lu L., Gao D., Liu Z., Zhao F. (2023). Review: Application of chitosan and its derivatives in medical materials. Int. J. Biol. Macromol..

[4] Sharma A., Kaur I., Dheer D., Nagpal M., Kumar P., Venkatesh D.N., Puri V., Singh I. (2023). A propitious role of marine sourced polysaccharides: Drug delivery and biomedical applications. Carbohydr. Polym..

[5] Samrot A.V., Sathiyasree M., Rahim S.B.A., Renitta R.E., Kasipandian K., Shree S.K., Rajalakshmi D., Shobana N., Dhiva S., Abirami S. (2023). Scaffold using chitosan, agarose, cellulose, dextran and protein for tissue engineering—A review. Polymers.

[6] Wang Y., Liu K., Zhang M., Xu T., Du H., Pang B., Si C. (2023). Sustainable polysaccharide-based materials for intelligent packaging. Carbohydr. Polym..

[7] Ghiorghita C.-A., Dinu M.V., Lazar M.M., Dragan E.S. (2022). Polysaccharide-based composite hydrogels as sustainable materials for removal of pollutants from wastewater. Molecules.

[8] Lazar M.M., Ghiorghita C.-A., Dragan E.S., Humelnicu D., Dinu M.V. (2023). Ion-imprinted polymeric materials for selective adsorption of heavy metal ions from aqueous solution. Molecules.

[9] Abdallah M.M., Fernández N., Matias A.A., Bronze do Rosário M. (2020). Hyaluronic acid and chondroitin sulfate from marine and terrestrial sources: Extraction and purification methods. Carbohydr. Polym.

[10] Dinu M.V., Lazar M.M., Ghiorghita C.-A., Raschip I.E., Dodda J.M., Deshmukh K., Bezuidenhout D. (2023). Multicomponent hydrogels for controlled drug release and delivery. Multicomponent Hydrogels: Smart Materials for Biomedical Applications.

[11] Nayak A.K., Hasnain M.S., Aminabhavi T.M. (2021). Drug delivery using interpenetrating polymeric networks of natural polymers: A recent update. J. Drug Deliv. Sci. Technol..

[12] Soma P.K., Williams P.D., Lo Y.M. (2009). Advancements in non-starch polysaccharides research for frozen foods and microencapsulation of probiotics. Front. Chem. Eng. China.

[13] Raschip I.E., Fifere N., Dinu M.V., Roohi M.K. (2021). Polysaccharide-based materials as promising alternatives to synthetic-based plastics for food packaging applications. Bioplastics for Sustainable Development.

[14] Coria-Hernández J., Meléndez-Pérez R., Méndez-Albores A., Arjona-Román J.L. (2021). Effect of cryostructuring treatment on some properties of xanthan and karaya cryogels for food applications. Molecules.

[15] Huang J., Deng Y., Ren J., Chen G., Wang G., Wang F., Wu X. (2018). Novel in situ forming hydrogel based on xanthan and chitosan re-gelifying in liquids for local drug delivery. Carbohydr. Polym..

[16] Martın-Illana A., Chinarro E., Cazorla-Luna F., Notario-Perez R., Veiga-Ochoa M.D., Rubio J., Tamayo A. (2022). Optimized hydration dynamics in mucoadhesive xanthan-based trilayer vaginal films for the controlled release of tenofovir. Carbohydr. Polym..

[17] Hanna D.H., Saad G.R. (2019). Encapsulation of ciprofloxacin within modified xanthan gum-chitosan based hydrogel for drug delivery. Bioorg. Chem..

[18] Bhattacharya S.S., Shukla S., Banerjee S., Chowdhury P., Chakraborty P., Ghosh A. (2013). Tailored IPN hydrogel bead of sodium carboxymethyl cellulose and sodium carboxymethyl xanthan gum for controlled delivery of diclofenac sodium. Polym. Plast. Technol. Eng..

[19] Biswas A., Mondal S., Das S.K., Bose A., Thomas S., Ghosal K., Roy S., Provaznik I. (2021). Development and characterization of natural product derived macromolecules based interpenetrating polymer network for therapeutic drug targeting. ACS Omega.

[20] Sethi S., Kaith S.B.S., Kaur M., Sharma N., Kumar V. (2020). Cross-linked xanthan gum–starch hydrogels as promising materials for controlled drug delivery. Cellulose.

[21] Raschip I.E., Hitruc E.G., Oprea A.M., Popescu M.C., Vasile C. (2011). In vitro evaluation of the mixed xanthan/lignin hydrogels as vanillin carriers. J. Mol. Struct..

[22] Raschip I.E., Paduraru-Mocanu O.M., Nita L.E., Dinu M.V. (2020). Antibacterial porous xanthan-based films containing flavoring agents evaluated by near infrared chemical imaging technique. J. Appl. Polym. Sci..

[23] Raschip I.E., Fifere N., Varganici C.-D., Dinu M.V. (2020). Development of antioxidant and antimicrobial xanthan-based cryogels with tuned porous morphology and controlled swelling features. Int. J. Biol. Macromol..

[24] Raschip I.E., Dinu M.V., Fifere N., Darie-Nita R.N., Pamfil D., Popirda A., Logigan C. (2020). Mechanical, thermal and surface properties of novel xanthan-based cryogels. Cellul. Chem. Technol..

[25] Raschip I.E., Fifere N., Dinu M.V. (2021). A comparative analysis on the effect of variety of grape pomace extracts on the ice-templated 3D cryogel features. Gels.

[26] Anwar M., Pervaiz F., Shoukat H., Noreen S., Shabbir K., Majeed A., Ijaz S. (2021). Formulation and evaluation of interpenetrating network of xanthan gum and polyvinylpyrrolidone as a hydrophilic matrix for controlled drug delivery system. Polym. Bull..

[27] Pervaiz F., Tanveer W., Shoukat H., Rehman S. (2023). Formulation and evaluation of polyethylene glycol/Xanthan gum-co-poly (Acrylic acid) interpenetrating network for controlled release of venlafaxine. Polym. Bull..

[28] Giannouli P., Morris E.R. (2003). Cryogelation of xanthan. Food Hydrocoll..

[29] Raschip I.E., Darie-Nita R.N., Fifere N., Hitruc G.-E., Dinu M.V. (2023). Correlation between mechanical and morphological properties of polyphenol-laden xanthan gum/poly(vinyl alcohol) composite cryogels. Gels.

[30] Zhang H., Zhang F., Wu J. (2013). Physically crosslinked hydrogels from polysaccharides prepared by freeze–thaw technique. React. Funct. Polym..

[31] Lozinsky V.I. (2002). Cryogels on the basis of natural and synthetic polymers: Preparation, properties and application. Russ. Chem. Rev..

[32] Zhang H., Liu C., Chen L., Dai B. (2019). Control of ice crystal growth and its effect on porous structure of chitosan cryogels. Chem. Eng. Sci..

[33] Lozinsky V.I. (2018). Cryostructuring of polymeric systems. 50. Cryogels and cryotropic gel-formation: Terms and definitions. Gels.

[34] Selin S.S., Sahin D., Berkant Y., Rawil F., Ekaterina N., Oguz O., Ramesh S.A., Nurettin S. (2019). Cryogel composites based on hyaluronic acid and halloysite nanotubes as scaffold for tissue engineering. Int. J. Biol. Macromol..

[35] Corman M.E. (2018). Poly-L-lysine modified cryogels for efficient bilirubin removal from human plasma. Colloids Surf. B.

[36] Hixon K.R., Lu T., Sell S.A. (2017). A comprehensive review of cryogels and their roles in tissue engineering applications. Acta Biomater..

[37] Memic A., Colombani T., Eggermont L.J., Rezaeeyazdi M., Steingold J., Rogers Z.J., Navare K.J., Mohammed H.S., Bencherif S.A. (2019). Latest advances in cryogel technology for biomedical applications. Adv. Therap..

[38] Serex L., Braschler T., Filippova A., Rochat A., Béduer A., Bertsch A., Renaud P. (2018). Pore size manipulation in 3D printed cryogels enables selective cell seeding. Adv. Mater. Technol..

[39] Baimenov A.Z., Berillo D.A., Moustakas K., Inglezakis V.J. (2020). Efficient removal of mercury (II) from water by use of cryogels and comparison to commercial adsorbents under environmentally relevant conditions. J. Hazard. Mater..

[40] Ma S., Li Y., Ma C., Wang Y., Ou J., Ye M. (2019). Challenges and advances in the fabrication of monolithic bioseparation materials and their applications in proteomics research. Adv. Mater..

[41] Chen Y., Zhang L., Yang Y., Pang B., Xu W., Duan G., Jiang S., Zhang K. (2021). Recent progress on nanocellulose aerogels: Preparation, modification, composite fabrication, applications. Adv. Mater..

[42] Sharma M., Tavares A.P.M., Nunes J.C.F., Singh N., Mondal D., Neves M.C., Prasad K., Freire M.G. (2020). Hybrid alginate-protein cryogel beads: Efficient and sustainable bio-based materials to purify immunoglobulin G antibodies. Green Chem..

[43] Tang C., Brodie P., Brunsting M., Tam K.C. (2020). Carboxylated cellulose cryogel beads via a one-step ester crosslinking of maleic anhydride for copper ions removal. Carbohydr. Polym..

[44] Tavsanli B., Okay O. (2020). Macroporous methacrylated hyaluronic acid cryogels of high mechanical strength and flow-dependent viscoelasticity. Carbohydr. Polym..

[45] Dragan E.S., Humelnicu D., Dinu M.V. (2021). Designing smart triple-network cationic cryogels with outstanding efficiency and selectivity for deep cleaning of phosphate. Chem. Eng. J..

[46] Pencheva V., Margaritova E., Borinarova M., Slavkova M., Momekova D., Petrov P.D. (2018). A novel approach for fabricating nanocomposite materials by embedding stabilized core-shell micelles into polysaccharide cryogel matrix. Carbohydr. Polym..

[47] Zheng M., Lian F., Xiong Y., Liu B., Zhu Y., Miao S., Zhang L., Zheng B. (2019). The synthesis and characterization of a xanthan gum-acrylamide trimethylolpropane triglycidyl ether hydrogel. Food Chem..

[48] Kang M., Oderinde O., Liu S., Huang Q., Ma W., Yao F., Fu G. (2019). Characterization of Xanthan gum-based hydrogel with Fe^3+^ ions coordination and its reversible sol-gel conversion. Carbohydr. Polym..

[49] Zhang Q., Hu X.M., Wu M.Y., Wang M.M., Zhao Y.Y., Li T.T. (2019). Synthesis and performance characterization of poly(vinyl alcohol)-xanthan gum composite hydrogel. React. Funct. Polym..

[50] Brunchi C.-E., Bercea M., Morariu S., Avadanei M. (2016). Investigations on the interactions between xanthan gum and poly(vinyl alcohol) in solid state and aqueous solutions. Eur. Polym. J..

[51] Elella M.H.A., Sabaa M.W., El Hafeez E.A., Mohamed R.R. (2019). Crystal violet dye removal using cross-linked grafted xanthan gum. Int. J. Biol. Macromol..

[52] Seright R.S., Henrici B.J. (1990). Xanthan stability at elevated temperatures. SPE Res. Eng..

[53] Krstonošić V., Milanović M., Dokić L. (2019). Application of different techniques in the determination of xanthan gum-SDS and xanthan gum-Tween 80 interaction. Food Hydrocoll..

[54] Dinu M.V., Gradinaru A.C., Lazar M.M., Dinu I.A., Raschip I.E., Ciocarlan N., Aprotosoaie A.C. (2021). Physically cross-linked chitosan/dextrin cryogels entrapping *Thymus vulgaris* essential oil with enhanced mechanical, antioxidant and antifungal properties. Int. J. Biol. Macromol..

[55] Raschip I.E., Hitruc G.E., Vasile C., Popescu M.-C. (2013). Effect of the lignin type on the morphology and thermal properties of the xanthan/lignin hydrogels. Int. J. Biol. Macromol..

